# Three-dimensional visualization of the vascular bundle in a branched bamboo node

**DOI:** 10.3389/fpls.2023.1256772

**Published:** 2023-10-25

**Authors:** Shan Li, Qianying Yang, Yangao Wang, Lili Shang, Shumin Yang, Xing’e Liu, Qianli Ma, Zixiong Cao

**Affiliations:** ^1^ Department of Biomaterials, International Centre for Bamboo and Rattan, Beijing, China; ^2^ Key Laboratory of National Forestry and Grassland Administration, Beijing for Bamboo & Rattan Science and Technology, Beijing, China; ^3^ College of Forestry, Sichuan Agricultural University, Chengdu, China; ^4^ Application Support Team, Object Research Systems (ORS), Inc., Montréal, QC, Canada

**Keywords:** bamboo, branched node, deep-learning algorithms, three-dimensional reconstruction, vascular bundle, X-ray microtomography

## Abstract

Bamboo is a natural vascular bundle (VB) reinforced composite material used in more than 10 fields such as construction and furniture. The nodes in bamboo are crucial to its mechanical properties, but understanding of its performance is limited by lack of knowledge about the three-dimensional (3D) structure of the node. This work aimed to non-destructively identify the multi-dimensional characteristics of the VB in a bamboo branched node (BN) using X-ray microtomography (µCT). The VB was segmented from the BN using deep learning combined with the Watershed algorithm. The 3D model reconstruction and characterization of the VB were also conducted. It was found that the structure of VBs showed significant changes along the height of the BN. The VBs formed a complex 3D structure, VBs of the culm are connected with those of the branch, and the connectivity of the conducting tissue and fibers was 88.91% and 99.95%, respectively. The conducting tissue and the fibers had similar shapes but varying thicknesses, which enabled VBs to perform both water transport and mechanical support functions. The volumes fraction of parenchyma, fibers, and conducting tissue in the BN were 61.3%, 35.3%, and 3.4%, respectively, but the tissue proportion of the different heights of the BN varied from each other. The nodal ridge was a mechanical weak point of the BN, with a maximum fibers proportion of 43.8%. This study contributes to understanding the relationship of VBs between the branch and the culm. It provides a structural perspective for understanding the mechanical properties of BN and a theoretical basis for optimizing bamboo utilization efficiency.

## 1 Introduction

Bamboo is a rapidly growing plant that significantly contributes to human life and productivity in various industries, including construction, furniture, papermaking, etc., because of its excellent physical and mechanical properties (Khalil et al., 2012; Sun et al., 2015; Song et al., 2017; Fang et al., 2018; Chen et al., 2020). Notably, nodes are a fundamental feature of bamboo and are one of the factors that determine bamboo’s performance (Kappel et al., 2004; Shao et al., 2010a; Taylor et al., 2015). The node has a reinforcing effect on the bending strength, compressive strength, energy absorption, and flexibility of the bamboo culm (Lee and Liu, 2003; Wegst, 2008; Zou et al., 2018), but reduce the uniformity, elasticity, buckling resistance, and tensile strength of bamboo (Hamdan et al., 2009; Wang et al., 2012; Cui et al., 2020). There is a lack of sufficient cognition of the relationship between the structure and mechanical properties because of the insufficient understanding of the structure of bamboo nodes. Therefore, it is necessary to conduct research on the structure of bamboo nodes.

The structure of bamboo node has a significant structural difference from the internode (Shao et al., 2010b; Zhang et al., 2017a), althrough they are all composed of vascular bundles (VBs) and parenchyma. The VB plays a key role in mechanical properties of bamboo (Wang et al., 2014; Zou et al., 2016; Palombini et al., 2020), mainly affected by its morphological character, quantity, distribution, and tissue proportion of VBs (Xian and Xian, 1990; Amada and Untao, 2001; Ghavami et al., 2003; Palombini et al., 2020). However, the intricated arrangement of VBs in the bamboo nodes, which significantly influence the bamboo structure, remains largely unknown, especially in the branched nodes (BNs). The structural features of the bamboo node have been previously explored using natural decay and tissue sectioning with a microscope (Grosser and Liese, 1971; Xiong et al., 1980; Ding and Liese, 1995; Ding et al., 2000). However, these methods are limited because they are time-consuming, and samples are easily damaged, leading to inaccurate results. Moreover, the accuracy of two-dimensional (2D) image characterization techniques is associated with the location and angle of the specimen. A three-dimensional (3D) higher-resolution non-destructive testing method is thus a more suitable characterization technique.

X-ray microtomography (µCT) is an ideal non-destructive testing equipment for studying the VB in a BN because of its high spatial resolution and intuitive results (Landis and Keane, 2010). It can also obtain serial sections of the specimen in any direction, thus advantageous in studying the anatomical structure. µCT applied to study the bamboo node enables visualization of the anatomical structure, 3D visualization, quantitative characterization, and numerical simulation (Palombini et al., 2020; Li et al., 2021; Xiang et al., 2021). Peng et al. (2014) and Xiang et al. (2021) used µCT to study the complex VB network of bamboo nodes but did not separately extract the VB to observe its morphology, thus lacking intuitive visualization. Huang et al. analyzed the density distribution variability of the bamboo node in different directions and their porosity based on 2D images (Huang et al., 2015; Huang et al., 2017). The development of image processing technology has enabled the separation of VBs from the bamboo node and the 3D visualization of its morphology. Palombini et al. (2020) showed that branches from the basal portion to the branching were continuous, without bridges or vascular traces, and were connected with the culm. Li et al. (2021) reported that the VB of the unbranched node formed an intricate and highly connected 3D network structure. The morphology and direction of the bamboo VB changed greatly in the node (Li et al., 2021) because of the branch (Xiang et al., 2021). The VB arrangement in the node and the branch of the BN thus remains unclear. How the branch impacts the node’s structure in the different bamboo node heights also remains unclear. The bamboo node branch is thus an important feature that requires a comprehensive study.

This study aimed to obtain the VB structure in a BN of *Chimonobambusa tumidissinoda* Hsueh & T. P. Yi ex Ohrnberger bamboo in a fast, non-destructive, and accurate manner using high-resolution µCT. The study further aimed to reconstruct a 3D model of the conducting tissue and fibers of the BN, explore the structural relationship between the culm and the branch, and discuss the relationship between structure and performance of the BN. This study contributes to the existing understanding of the structure of the bamboo BN.

## 2 Materials and methods

### 2.1 Materials

**Figure 1:**
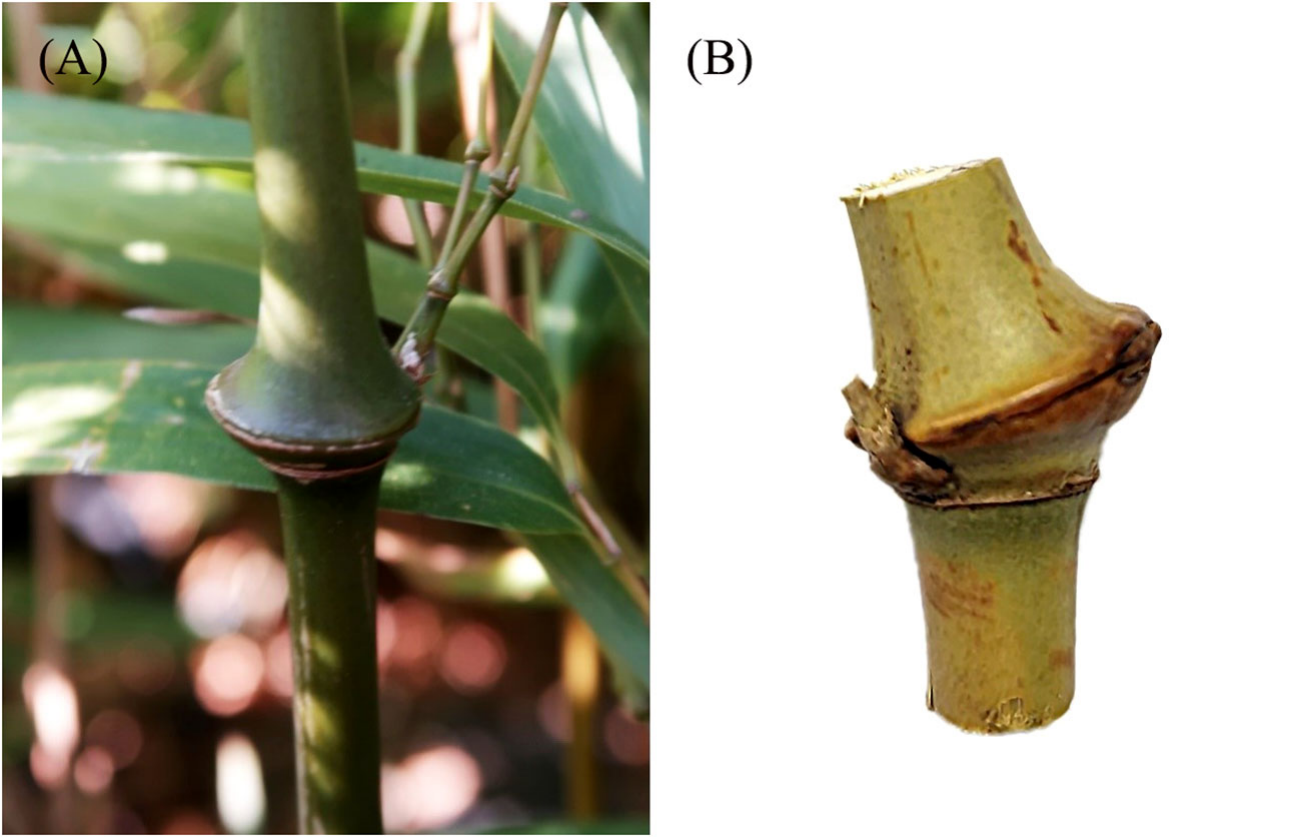
Sample preparation for μCT analysis: **(A)** *Chimonobambusa tumidissinoda* bamboo; **(B)** A complete BN excised from the bamboo culm for μCT analysis.

Five 2-year-old *C. tumidissinoda* bamboos (**Figure 1A**) were cut from Daguan County, Yunnan, P. R. China, and transported to the laboratory. More than ten bamboo nodes performed µCT scanning, including nodes with a single branch, nodes without branches, and nodes with three branches (**Supplementary Figure 1**). These bamboo nodes were used to observe anatomical structure. A smaller and anatomically representative BN was selected for the subsequent analysis (**Figure 1B**). This bamboo node with a diameter of 1.6 cm was removed from the third node of a bamboo culm that was 1.3 m in height, at a height located 20 cm from the base, following natural air drying with a moisture content of 3.46%. The length of the internode below this node was 9 cm, and the diameter was 0.8 cm. This selection was motivated by the fact that placing the entire BN within the scanning field of view allowed the smaller BN to achieve enhanced resolution, thereby facilitating the acquisition of finer structural details of the bamboo, the height of the bamboo node in the imaging area was 0.97 cm.

### 2.2 µCT imaging

The 3D imaging of bamboo nodes were performed at Skyscan2214 (Bruker, MA, USA). The parameter of µCT scanning were closely correlated with properties such as the size and density of the samples. The imaging parameters for the sample used in the analysis were: an X-ray source voltage of 70 kV, tube current of 130 µA, power of 2.04 W, rotation step of 0.1°, and exposure time of 108 ms, with each pixel representing a linear resolution of 7.5 μm. There were 2197 projections acquired during the 5 h 20 min. They were exported in TIFF format to allow further analyses.

### 2.3 Three-dimensional reconstruction and image segmentation

**Figure 2:**
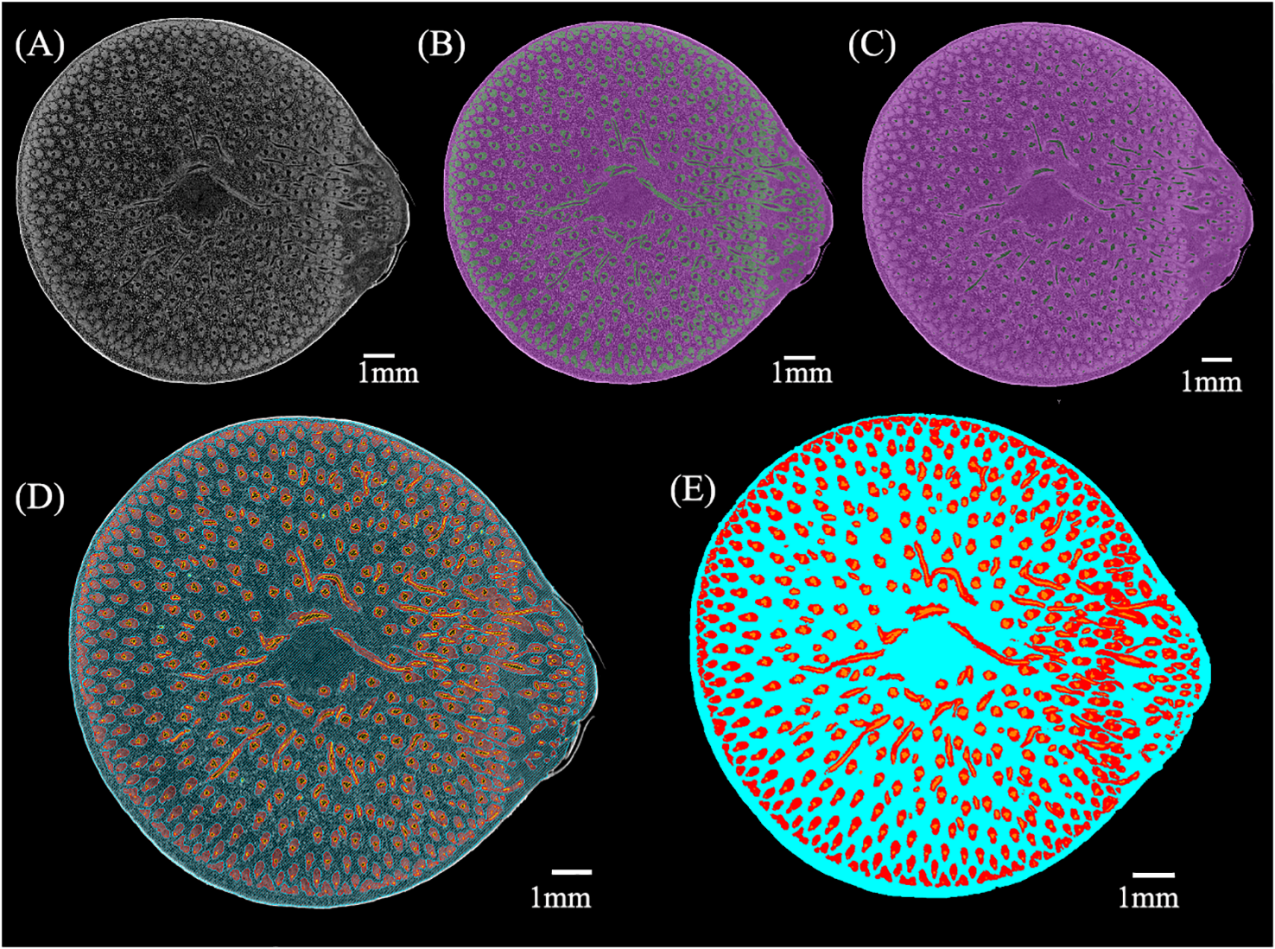
The process of µCT images segmentation: **(A)** Original µCT slice; **(B)** Deep learning segmentation of fibers (green) and others (purple); **(C)** Deep learning segmentation of conducting tissue (green) and others (purple); **(D)** Watershed segmentation of the BN (conducting tissue is orange, fibers are red, parenchyma is blue); **(E)** Pseudo-color map of conducting tissue (orange), fibers (red), and parenchyma (blue).

The projection images were reconstructed using a noniterative Filtered Backprojection algorithm in the NRecon software. A sequence of slices was acquired and exported as a stack of TIFF images, obtaining 1293 slices with serial numbers 0-1292 (**Figure 2A**). The thickness of the conducting tissue and fibers in the VB of the bamboo node changed in the opposite pattern (Li et al., 2021). The conducting tissue and fibers were segmented to better visualize and quantify the VB.

µCT images were segmented using the deep learning U-Net model and manual method in the Dragonfly software (Object Research Systems, Montreal, QC, Canada), besides the segmentation done using the Watershed algorithm in the Avizo software (FEI Hillsboro, OR, USA). Automatic and manual methods were used to segment the µCT images as follows:

(1) Fibers (**Figure 2B**) and conducting tissue (**Figure 2C**) were segmented from the BN using deep learning in the Dragonfly software.
(2) The segmentation results of (1) were manually checked.
(3) The watershed method was applied to segment the BN from others in the µCT images. The branches in the slices above slice722 of the BN were not marked because of the culm separation. Then, two pixels of the segmentation of the BN were eroded to remove its cortex.
(4) The conducting tissue in the result of (2) was dilated to ensure no pores between the fibers and the conducting tissue.
(5) Parenchyma was calculated by subtracting fibers in the result of (2) and conducting tissue in the result of (4) from the BN in the result of (3).
(6) The sum of pixel intersections by each two of the fibers and the conducting tissue in the result of (2) and the parenchyma in the result of (5) were removed.
(7) The fibers, conducting tissue, and parenchyma in the result of (6) were used as the initial seed spot to perform watershed segmentation (**Figure 2D**).

The pseudo-color map of different tissues was obtained (**Figure 2E**), allowing further analyses.

### 2.4 Statistical analysis

The size of axial vascular bundle (AVBs) was measured by assessing the length and width of AVBs located within the cross-shaped marquees (2.94×30.52 mm^2^, 30.64×3.16 mm^2^) designated on each slice (Slices 0, 50, 110, 300, 385, 600, 872, and 1292). The significant analysis of the variance of the AVB’s size was subjected to Duncan’s test using the IBM SPSS Statistics 19 software program. The number of VBs was counted on slices 0, 50, 110, 300, 385, 600, 872, and 1292 in the ImageJ software. The area of the BN’s cross-section was measured using the AVIZO software. The frequency of the VB was the number of VBs to the area of the BN’s cross-section. The AVIZO software was also used to obtain the proportion, area, and volume of each type of tissue in the BN. These data were analyzed statistically and plotted using Origin 2018.

## 3 Results and discussion

### 3.1 Morphology of the bamboo BN

#### 3.1.1 Macrostructure and tissues constitution

**Figure 3:**
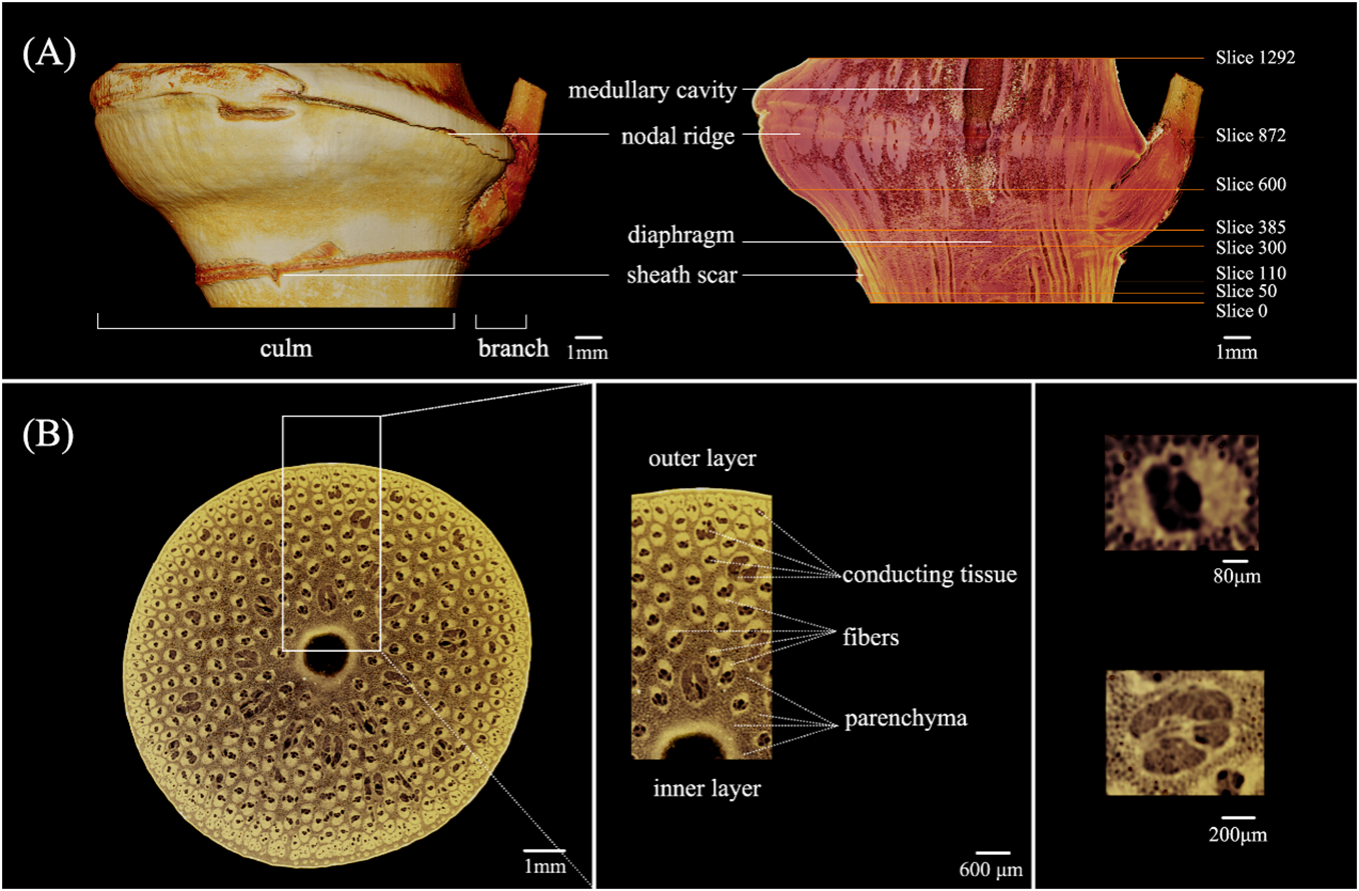
Morphology of the *Chimonobambusa tumidissinoda* node: **(A)** Exterior view and longitudinal section; **(B)** The cross-sectional view and details.

The morphology features of the *C. tumidissinoda* BN were shown in **Figure 3A**. The BN had a culm and a branch, the branch was between the sheath scar and the nodal ridge, the medullary cavity appeared under the nodal ridge. The junction of the branch and the culm was in a similar position as the diaphragm in height. The shape of unbranched side was more exoconvex than that of the culm side.

The morphological variations of the VB observed in the longitudinal section of the BN can be attributed to its transforming orientation and size (**Figure 3A**). In contrast, the internodal VBs appear to align nearly parallel to each other (Yu et al., 2002; Zhang et al., 2017b). Slices 0 - 1292 corresponded to the cross-section of the BN from the bottom to the top. Slices 50, 110, 300, 385, 600, and 872 were on the lower end of the diaphragm, near the sheath scar, the upper end of the diaphragm, medullary cavity, the lower end of the nodal ridge, and nodal ridge, respectively. The obvious morphological changes of the VB in these slices allowed further analyses.

In the same line, the boundary of each tissue in the cross-section of the BN was clear, allowing the distinct observation of parenchyma, fibers, and conducting tissue. This finding suggested that µCT could achieve good imaging results of BN in *C. tumidissinoda*. The shape of the VB changed significantly and differed from the typical VB types, including the metaxylem vessels, phloem with sieve tubes, and protoxylem (**Figures 3A, B**).

#### 3.1.2 Structural changes of VBs in the BN

**Figure 4:**
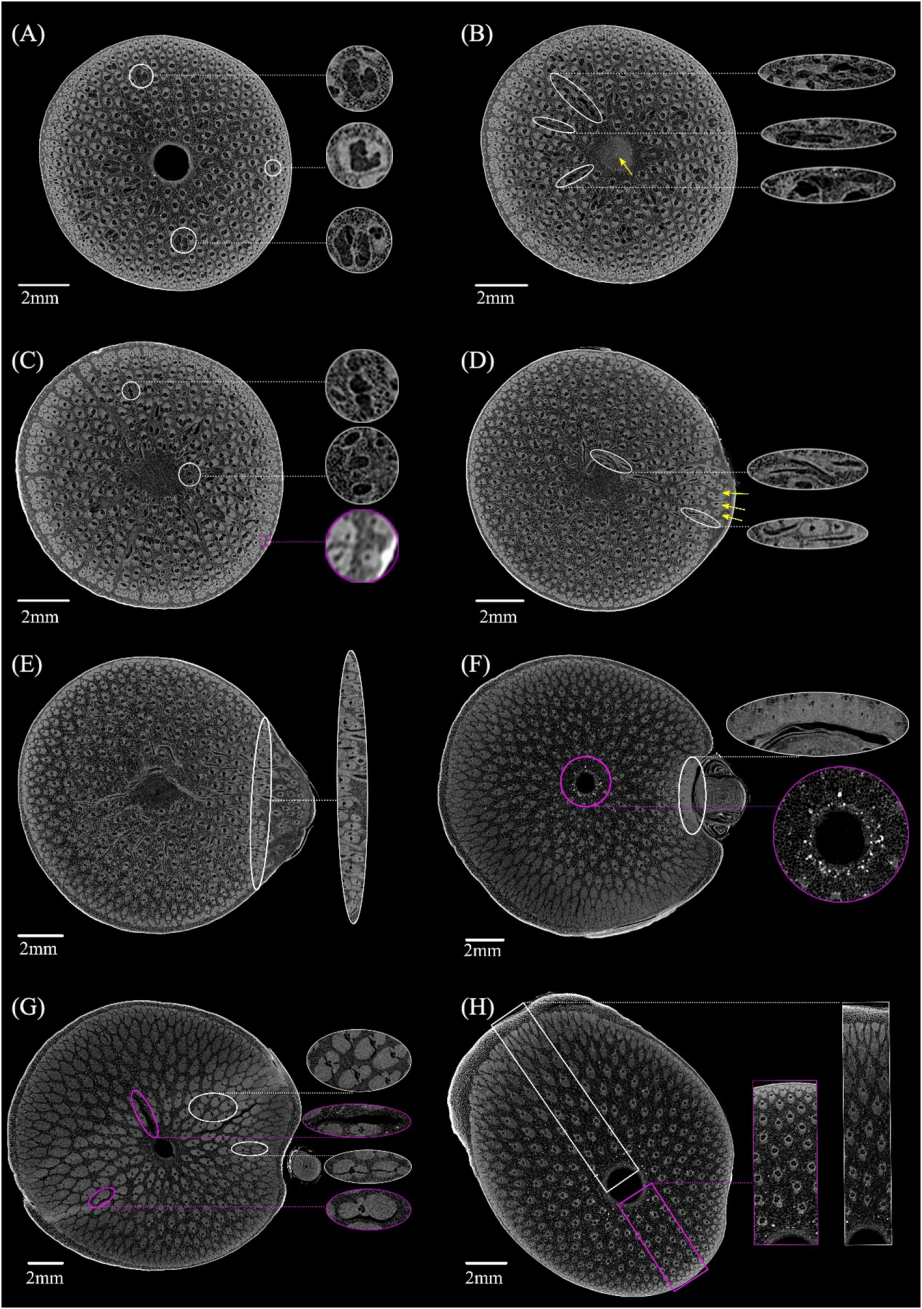
X-ray microtomography slices at different heights in the *Chimonobambusa tumidissinoda* node: **(A)** Slice 0: the VB with larger conducting tissue (white circle). **(B)** Slice 50: the TVB (white circle) and diaphragm (yellow arrow). **(C)** Slice110: the bifurcated small VB (white circle) and the small VB in the cortex (magenta circle). **(D)** Slice 300: branch (yellow arrow) and a TVB (white circle). **(E)** Slice 385: the boundary between the culm and the branch (white circle). **(F)** Slice 600: developed fibers near the branch of the culm and the cortex were not very pronounced (white circle), the starch granules (magenta circle). **(G)** Slice 872: shorter cracks (white circle) and longer cracks (magenta circle). **(H)** Slice 1292: inconsistent radial variation pattern of the VB (white box, magenta box).

The anatomical features at different heights in the BN was shown in **Figure 4**. Notable morphological variations were observed in the VB in cross-section along the vertical direction of the BN. Some VBs were present at the bottom of the BN but lacked the characteristic features of typical VBs based on the shape of their fibers. The metaxylem vessels and sieve tubes displayed an absence of a clearly demarcated boundary and amalgamated into a larger conducting tissue in VBs. (**Figure 4A**, white circle). Some VBs changed direction on the lower end of the diaphragm, appearing as a transverse vascular bundle (TVB) in the cross-section (**Figure 4B**, white circle). Notably, the image of the diaphragm at this location is relatively bright (**Figure 4B**, yellow arrow). In the same line, some small VBs were found near the sheath scar in the cortex (**Figure 4C**, magenta circle), while others were located in the culm of the BN, bifurcated from the VB (**Figure 4C**, white circle). Branching emerged at the node on the upper end of the diaphragm (**Figure 4D**, yellow arrow), while the TVB was predominantly located on the side of the culm close to the branch (**Figure 4D**, white circle). However, a significant amount of TVBs were distributed at the diaphragm of the unbranched bamboo node (Li et al., 2021). This indicates that the branch had an impact on the distribution of TVBs in the BN. The diaphragm disappeared under the nodal ridge, but numerous TVBs remained interconnected through rotation (**Figure 4E**). The boundary between the culm and the branch was apparent and was mainly characterized by abundant VBs (**Figure 4E**, white circle). The diaphragm disappeared, the culm and branch of the bamboo node separated, and the fibers near the branch side were particularly developed and connected together, which is different from the result of the previous study that the fibers of unbranched bamboo nodes were distributed more evenly in the cross-section under the nodal ridge (Li et al., 2021). Further research is needed to explore the impact of this uneven fiber distribution on the BN properties. The cortex was not very pronounced on the sides of the culm closer to the branch (**Figure 4F**, white circle), and some starch granules appeared near the medullary cavity at the lower end of the nodal ridge (**Figure 4F**, magenta circle). The sudden enlargement of the nodal ridge resulted in cracking, which could be categorized into two: shorter cracks found at the interface between fibers and conducting tissue (**Figure 4G**, white circle) and longer cracks at the interface between fibers and parenchyma (**Figure 4G**, magenta circle). These observations suggested that the interface between different tissues and the point of abrupt node enlargement were the areas where the BN was prone to cracking. There was an inverse relationship among the radial variations of VB size within the same cross-section on the upper part of the BN. Specifically, on the swollen side of the BN, the size of the VB gradually decreased from the outer to the inner layer (**Figure 4H**, white box) but increased gradually from the outer to the inner layer on the other side (**Figure 4H**, magenta box). This phenomenon resembles the size distribution pattern of VBs at the top of unbranched bamboo nodes (Li et al., 2021). These findings added the conclusions drawn by other researchers, who only suggested that the VB size of the bamboo culm increases progressively from the outer to the inner layer (Kanzawa et al., 2011; Chen et al., 2018; Wang and Shao, 2020). The development of reaction wood is typically concomitant with eccentric growth (Liu et al., 2023). However, bamboo lacks vascular cambium and therefore does not exhibit reaction tissues associated with secondary growth (Ahmad and Kamke, 2005); as a result, this phenomenon in bamboo is not related to stress. By observing a large number of cross-sections of BN, the variations in anatomical characteristics of VB were captured at different heights. These results were difficult to achieve through microscopy techniques, which offer restricted spatial information (Grosser and Liese, 1971; Xiong et al., 1980; Ding and Liese, 1995; Ding et al., 2000), especially when dealing with the large sizes of TVBs and significant deviations in the transverse orientation of VBs.

### 3.2 Quantitative analysis of bamboo BN microstructure

#### 3.2.1 Size and quantity of AVBs

The size and quantity of AVBs at different height were quantitatively analyzed due to the structural changes of VBs along the BN height are obvious (**Table 1**). The size of the AVBs enlarged steadily from the bottom of the BN to the sheath scar. This trend was intricately linked with the amplification of conducting tissue within the AVBs in the region. Consequently, this enlargement slows down transpirational flow in the sheath scar, allowing nutrients to remain within the node for a longer period before reaching the branch (Yamaji and Ma, 2017). However, the size of the AVBs declined along the node from the sheath scar to the upper end of the diaphragm. This phenomenon was attributed to the fact that the AVB bifurcated some small VBs, reducing the size of the original one. Notably, the size of AVBs significantly increased from the point of the medullary cavity up to the nodal ridge, but decreased towards the top of the BN. The former effect is attributed to the disappearance of TVBs, whereas the latter is driven by the pronounced nodal morphology. The changes in VB size from the sheath scar to the diaphragm were inconsistent with the observations reported by the previous researchers (Li et al., 2021). This deviation may be attributed to the significant amount of TVBs in the branch.

**Table 1:** The analysis of variance of AVBs at different heights.

Slice position	BN position	radial length (mm)	Chord length (mm)
Slice 0	the bottom of the BN	0.43 ± 0.05^f^	0.27 ± 0.04^bcd^
Slice 50	the lower end of the diaphragm	0.45 ± 0.05^fg^	0.29 ± 0.03^d^
Slice110	near the sheath scar	0.47 ± 0.05^g^	0.29 ± 0.04^cd^
Slice 300	the upper end of the diaphragm	0.43 ± 0.06^f^	0.25 ± 0.04^ab^
Slice 385	the medullary cavity	0.44 ± 0.06^g^	0.24 ± 0.03^a^
Slice 600	the lower end of the nodal ridge	0.61 ± 0.17^h^	0.27 ± 0.05^bc^
Slice 872	the nodal ridge	0.81 ± 0.14^i^	0.40 ± 0.08^e^
Slice 1292	the top of the BN	0.47 ± 0.14^g^	0.27 ± 0.06^bcd^

No significant difference is observed between groups with the same letter in the table, and vice versa. (Duncan’s test, at 5% level).

The number and frequency of VBs in the slice of the BN varied with height (**Table 2**). The total count of VBs increased gradually from the bottom of the BN to the upper end of the diaphragm and decreased towards a stable level at the top of the BN. This phenomenon was attributed to the bifurcation of VBs between the lower end and the upper end of the diaphragm (Li et al., 2021). The number of AVBs decreased from the bottom of the node to the medullary cavity and then stabilized until the top of the BN. Notably, the part with reduced VBs corresponded with the emergence of the branch, whereas the position with stabilized VB number corresponded with the branch absence, indicating that the branch affected the number of VBs. The TVBs appeared in the lower end of the diaphragm and disappeared above the medullary cavity, with their number reaching the maximum at the upper end of the diaphragm. Moreover, the minimal size of the AVB in the upper end of the diaphragm indicated that an increased number of TVBs reduces the size of AVBs. The number of the branch VBs was less than those of the culm.

**Table 2:** The number and frequency of VBs in the slice at different heights of the BN.

Slice Position	BN Position	Number				Frequency	
		Culm		Branch	Total	Culm (/mm²)	Branch (/mm²)
		AVB	TVB				
Slice 0	the bottom of the BN	521			521	7.15	
Slice 50	the lower end of the diaphragm	519	17		536	6.87	
Slice110	near the sheath scar	514	40		554	6.62	
Slice 300	the upper end of the diaphragm	497	130		627	6.46	
Slice 385	the medullary cavity	495	108		603	5.39	
Slice 600	the lower end of the nodal ridge	496		66	562	3.21	18.9
Slice 872	the nodal ridge	499		63	562	2.67	30.8
Slice 1292	the top of the BN	497				3.63	

The frequency of the VB of the branch was significantly higher than that of the culm, confirming that the branching VBs were small and dense. The frequency of VBs in the culm decreased from the bottom of the BN to the nodal ridge, then increased towards the top of the BN. The frequency pattern of VBs was similar to that of the unbranched bamboo node (Li et al., 2021), with the minimum frequency of VBs located at the nodal ridge. The frequency of branch VBs increased from the bottom to the top in the BN, which was inconsistent with the culm regularity.

#### 3.2.2 Tissue proportion

**Figure 5:**
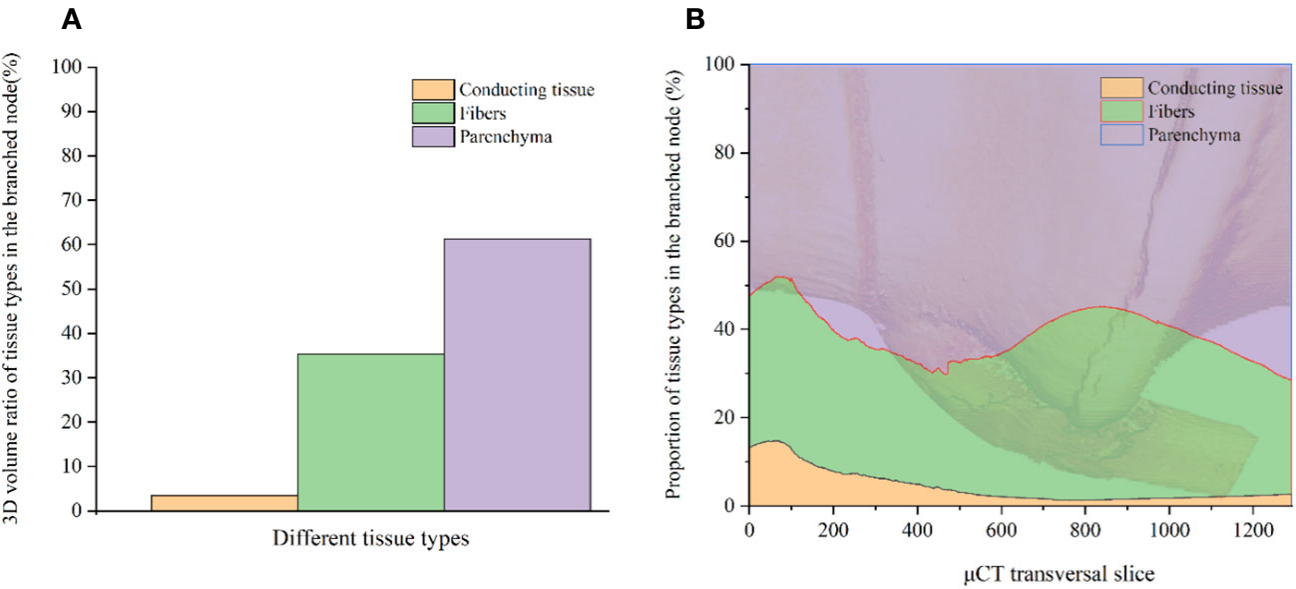
Tissue proportion in the BN: **(A)** The volume fraction of different tissues in the BN; **(B)** The proportional distribution of tissues in the BN.

An advantage of µCT scanning is its ability to analyze the 3D interior tissue proportions, enabling the acquisition of more representative volume data for tissues (**Figure 5A**) and highlighting changes across continuous node slices (**Figure 5B**). The proportion of tissue in each slice is quantified as the difference between the highest and lowest values corresponding to the respective colors. The volume fraction of parenchyma, fibers, and conducting tissue in the BN of *C. tumidissinoda* were 61.3%, 35.3%, and 3.4%, respectively (**Figure 5A**). However, there were continuous changes in tissue proportion at different heights from the bottom to the top of the BN (**Figure 5B**). The maximum proportion of parenchyma, fibers, and conducting tissue in the node are 71.5%, 43.8%, and 14.8%, respectively. These slices are located at the top of the BN, nodal ridge, and lower end of the diaphragm, respectively. The fibers in the nodal ridge exhibited the highest proportion in the BN (**Figure 5B**), which experiences high tensile and shear stresses at the junction of different internodes. Consequently, there is a greater abundance of fibers in this area to prevent culm fracture or bending. However, fiber cells are more prone to shrinkage under the influence of moisture content (Yuan et al., 2023). The abundant fibers in the nodal ridge is an important cause of macroscopic cracking in BN, which further leads to the BN becoming the weak point of bamboo. The minimum proportion of parenchyma, fibers, and conducting tissue in the node are 48.1%, 25.8%, and 1.3%, respectively. These slices are appearing in the lower end of the diaphragm, top of the node, and near the nodal ridge, respectively. The patterns of continuous changes in tissue proportion along the bamboo culms observed in this study are similar to those reported in previous studies (Li et al., 2021). However, the proportion of fibers in this study was higher, while that of parenchyma was lower because of node branching. These results further confirmed that the enlargement and bifurcation of the VB could lead to an increase in the proportion of the VB and a decrease in the parenchyma. The bifurcation of the VB caused an increase in the fibers and conducting tissue near the sheath scar, while enlargement of the VB caused an increase in the fibers and a decrease in the conducting tissue in the nodal ridge. The change patterns in fibers and conducting tissue thickness reported in this study are consistent with those reported in earlier studies (Li et al., 2021), which demonstrated inverse patterns between the thickness of conducting tissue and fibers at the nodal ridge.

### 3.3 Three-dimensional VB structure models

#### 3.3.1 Three-dimensional models of the conducting tissue

The anatomical characteristics showed the 2D information of the BN internal structure, which lacked the spatial structure and the continuous size variation related to the number and angle of the BN slices. A morphometrical study of the conducting tissue in a BN is instrumental in understanding its 3D features. Thickness is an important morphological index calculated as the diameter of a hypothetical sphere that fits within each boundary point using the Dragonfly software (Silin et al., 2011), where red denotes a larger thickness while purple denotes a smaller thickness.

**Figure 6:**
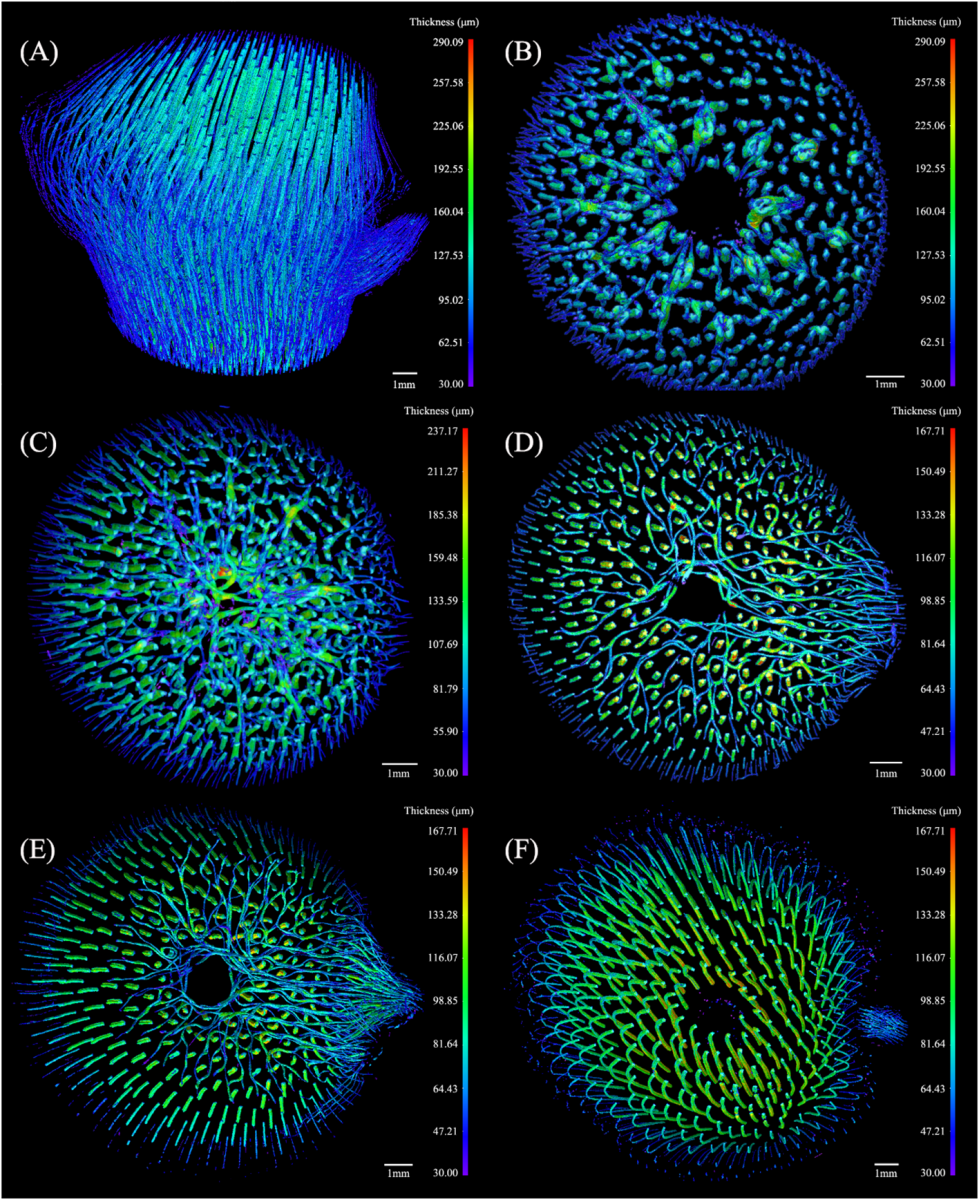
The 3D thickness model of the conducting tissue in the *Chimonobambusa tumidissinoda* BN: **(A)** BN; **(B)** Slices 0-110; **(C)** Slices111-300; **(D)** Slices 301-385; **(E)** Slices 386-600; **(F)** Slices 601-1292.

The thickness of the conducting tissue in the BN was shown in **Figure 6A**, with a maximum of 290.09 μm, revealing a slightly helical structure. The morphology of the conducting tissue in the segments was assessed (**Figures 6B–F**) to observe the variation of the conducting tissue at different heights of the BN. Parts of the conducting tissue under the sheath scar were bifurcated, forming the transverse conducting tissue (TCT). Notably, the thickness of the TCT was greater than that of the axial conducting tissue (ACT), that facilitates the transverse transport of water. The highest thickness of the conducting tissue was under the sheath scar (**Figure 6B**). The number of TCT increased while their thickness decreased (**Figure 6C**), the numerous bifurcated conducting tissue in the diaphragm facilitated the liquid movement in different directions. The TCT above the diaphragm was mainly congregated in the branch side of the BN, their thickness was less than that of the ACT (**Figure 6D**). In the lower end of the nodal ridge, the TCT gradually disappeared with the separation of the culm and branch, and some were distributed along the outer layer of the BN near the branch (**Figure 6E**). However, the TCT was not observed in the outer layer of the unbranched bamboo node (Li et al., 2021), which is related to the branch of the bamboo node. The unbranched conducting tissue above the branch changed slightly in direction, bending outward under the nodal ridge. The inward bend above the nodal ridge revealed a slightly helical structure (Palombini et al., 2016; Li et al., 2021). The thickness of the culm was greater than that of the branch (**Figure 6F**). The structure of the BN varied at different heights, unlike the internode structure of bamboo, which only exhibited a slight change (Yu et al., 2002; Zhang et al., 2017b). Unlike the unbranched bamboo nodes (Li et al., 2021), the TCT was not distributed in a concentric radial pattern. This work has revealed that the conducting tissue of the BN was more densely distributed on the branch side of the culm. Therefore, the conducting tissue structure of the culm would also be more biased toward new growth nutritional organs. In addition, although the TCT provided the possibility for transversal water transport, the thickness of the ACT decreased after the appearance of the TCT. This could reduce the axial water conductivity of the node.

**Figure 7:**
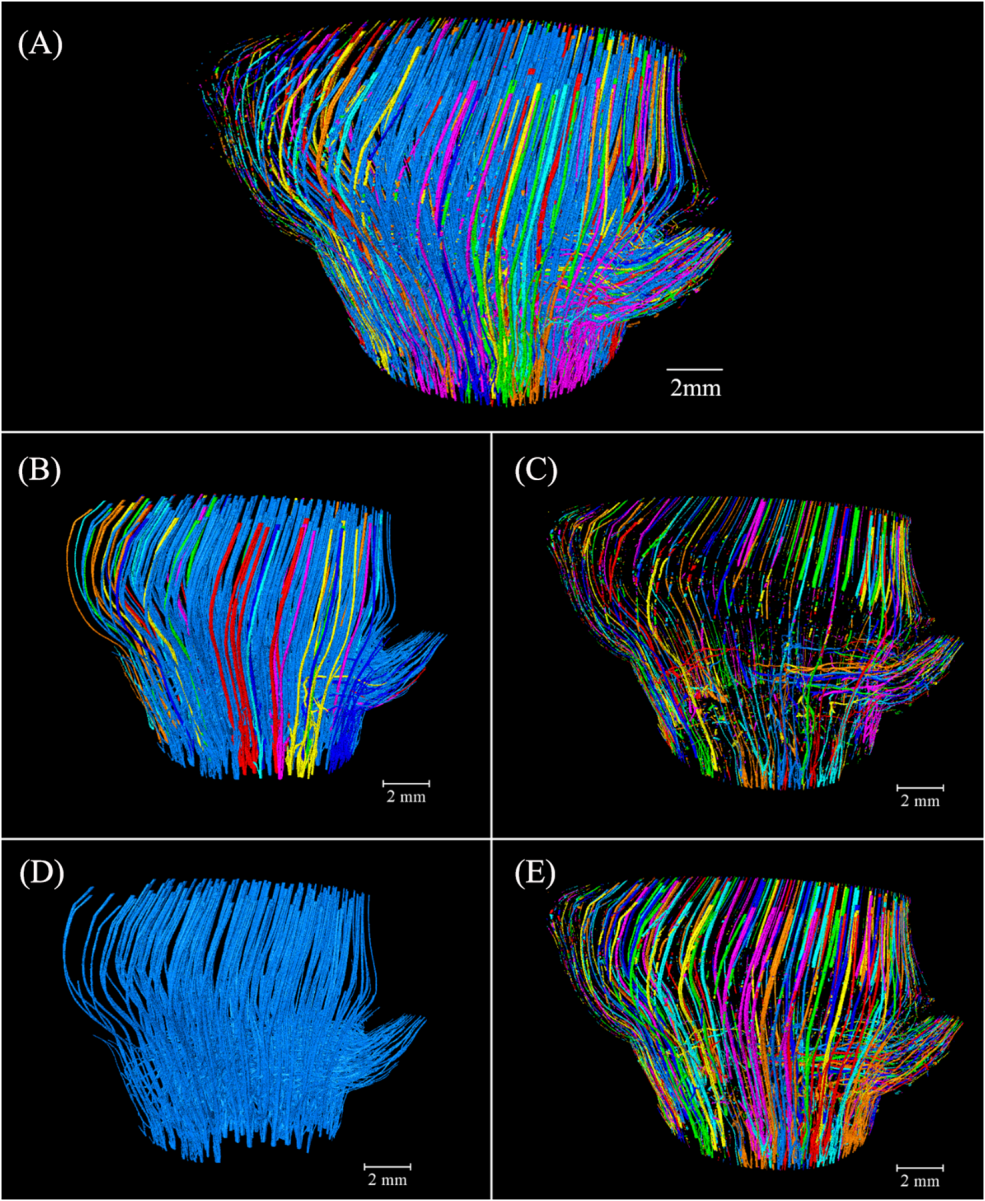
Spatial arrangement and orientation of the conducting tissue 3D model in *Chimonobambusa tumidissinoda* after label analysis: **(A)** The conducting tissue; **(B)** The connected conducting tissue; **(C)** The disconnected conducting tissue; **(D)** The largest conducting tissue volume; **(E)** The conducting tissue without the largest volume.

The 3D thickness model of the conducting tissue achieved a good observation of its morphological variation at different heights but did not elaborate on the relationship between the culm and branch. Image labeling provided valuable and advanced 3D insights into the intricate conducting tissue of the BN, distinguishing the volume of units by different colors (**Figure 7A**). The conducting tissue cannot be easily traced back to its origins (Kraehmer, 2017), connectivity of the conducting tissue originating from the Euler number is a fundamental topologic measure (Odgaard and Gundersen, 1993), which allows the characterization of the redundancy in structure connections (Bouxsein et al., 2010). The volume of the connected conducting tissue was 41.57 mm³ (**Figure 7B**). The connectivity in the conducting tissue with 46.76 mm³ was 88.91% (**Figure 7A**). The high connectivity of the conducting tissue in the 3D space revealed why VBs reportedly enter the diaphragm through elusive movement trails (Shao et al., 2010b; Chen et al., 2023). The disconnected conducting tissue was mostly distributed in the outer layer of the BN (**Figure 7C**). They were the unrecognizable small-sized conducting tissue and tissue bent directly into the unidentified branch above slice 722 observed during image processing. The largest conducting tissue volume, with 34.51 mm³, composed 73.81% of the total volume (**Figure 7D**). The conducting tissue of the culm and the branch were connected, forming an intricate network structure. This structure was distinct from the unconnected VBs of the culm and branch reported by Palombini et al. (2020). Some interconnected conducting tissue did not establish a connection with the largest conducting tissue (**Figure 7E**). This specific type of conducting tissue was primarily located within the outer layer of the BN. Investigating this phenomenon required the use of higher-resolution equipment. Visualization of fluid pathways within the bamboo BN was achieved by reconstructing a 3D model and analyzing the connectivity of the intersecting conducting tissue in the model. This work provided real evidence of the pathways of multidirectional fluid transport and the fluid pathways between the culm and the branch.

#### 3.3.2 Three-dimensional models of the fibers

**Figure 8:**
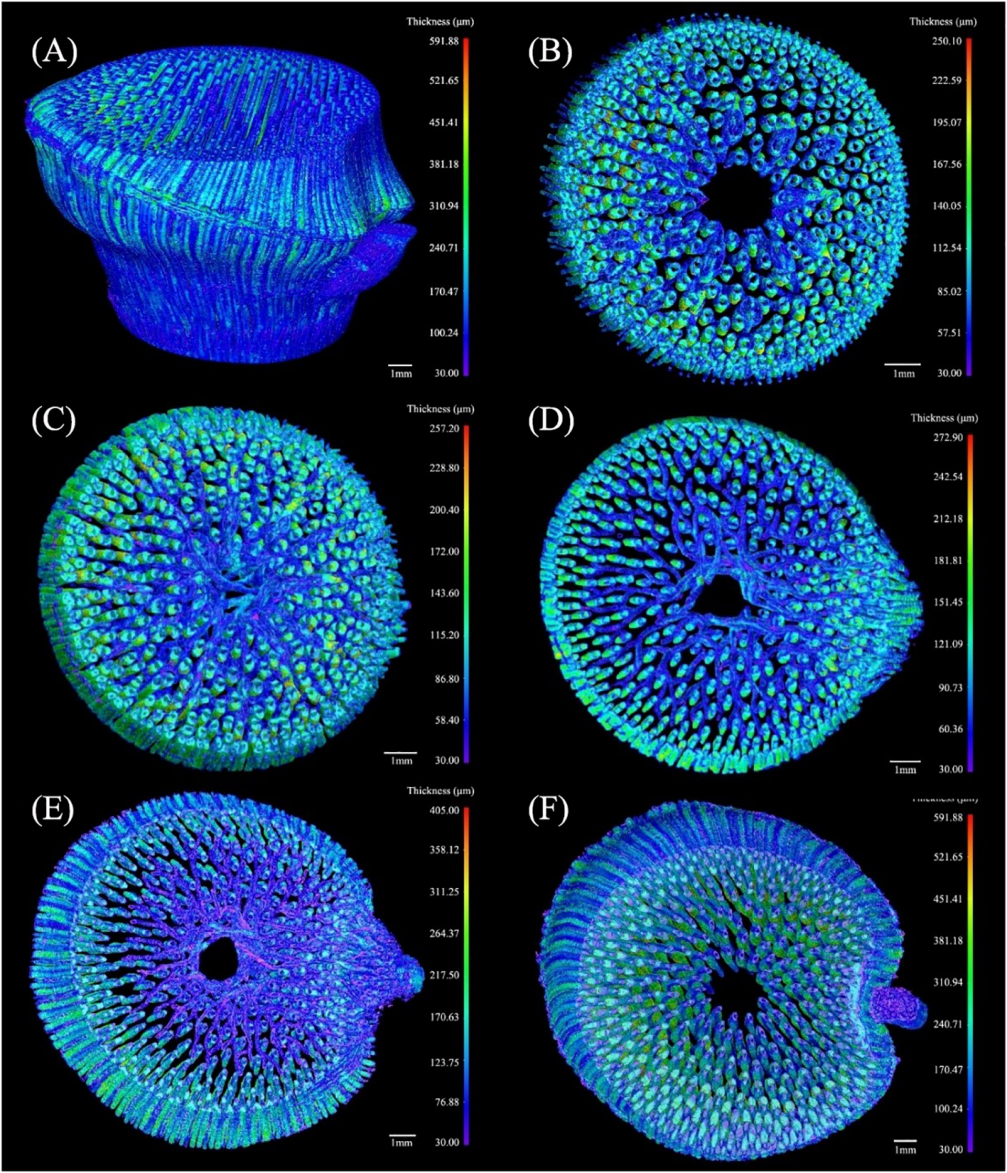
The 3D thickness model of the fibers in the branched *Chimonobambusa tumidissinoda* node: **(A)** BN; **(B)** Slices 0-110; **(C)** Slices111-300; **(D)** Slices 301-385; **(E)** Slices 386-600; **(F)** Slices 601-1292.

The thickness of the conducting tissue and fibers in the VB of the bamboo node were inversely correlated (Li et al., 2021). The fibers thickness was calculated similarly to that of the conducting tissue. The morphology of the fibers in the segments was examined to investigate the variations in their characteristics at various heights of the BN (**Figures 8B–F**).

The maximum thickness of fibers in the BN was 591.88 μm, which was greater than that of the conducting tissue. Here, the fibers were more dispersed while the conducting tissue was more concentrated. (**Figure 8A**). The thickness of the transverse fibers under the sheath scar was less than that of the axial fibers and was distinct from the regular pattern of the conducting tissue thickness (**Figure 8B**). The branching of fibers in the diaphragm was increased, forming a complex network structure (**Figure 8C**), which improved the node’s mechanical properties, including bending strength and splitting resistance (Zou et al., 2016; Huang et al., 2017). The range of fibers thickness above the diaphragm increased, especially on the branch side of the culm. This phenomenon was not observed in previous studies of unbranched bamboo nodes (Li et al., 2021), so it is inferred that the branch is accompanied by the appearance of a large number of fibers on the branch side of the culm, these fibers added structural support to the branch. The transverse fibers extended twistedly, possibly due to the squeezing between transverse and axial fibers (Chen et al., 2023). Notably, the thickness of the transverse fibers was less than that of the axial fibers (**Figure 8D**). This disparity was attributed to the requirement of axial fibers to support the weight of the entire bamboo plant, while transverse fibers are only responsible for supporting branches. Fibers in the lower end of the nodal ridge were mainly congregated in the branch side of the culm, similar to the distribution of conducting tissue (**Figure 8E**). However, fibers thickness from Slices 386 to Slices 600 increased, while that of the conducting tissue decreased, indicating that thin conducting tissue required more fibers for mechanical support and protection. Fibers in the Slices 601-1292 had the maximum thickness of the BN (**Figure 8F**), which was consistent with the location where the maximum proportion of fibers occurs. Nevertheless, conducting tissue and the fibers had similar shapes but varying thicknesses. The functional integration of mechanical reinforcement and liquid flow of the bamboo node was achieved through the thickness variations of their conducting tissue and fibers, showing the structural optimization potential of the bamboo nodes.

**Figure 9:**
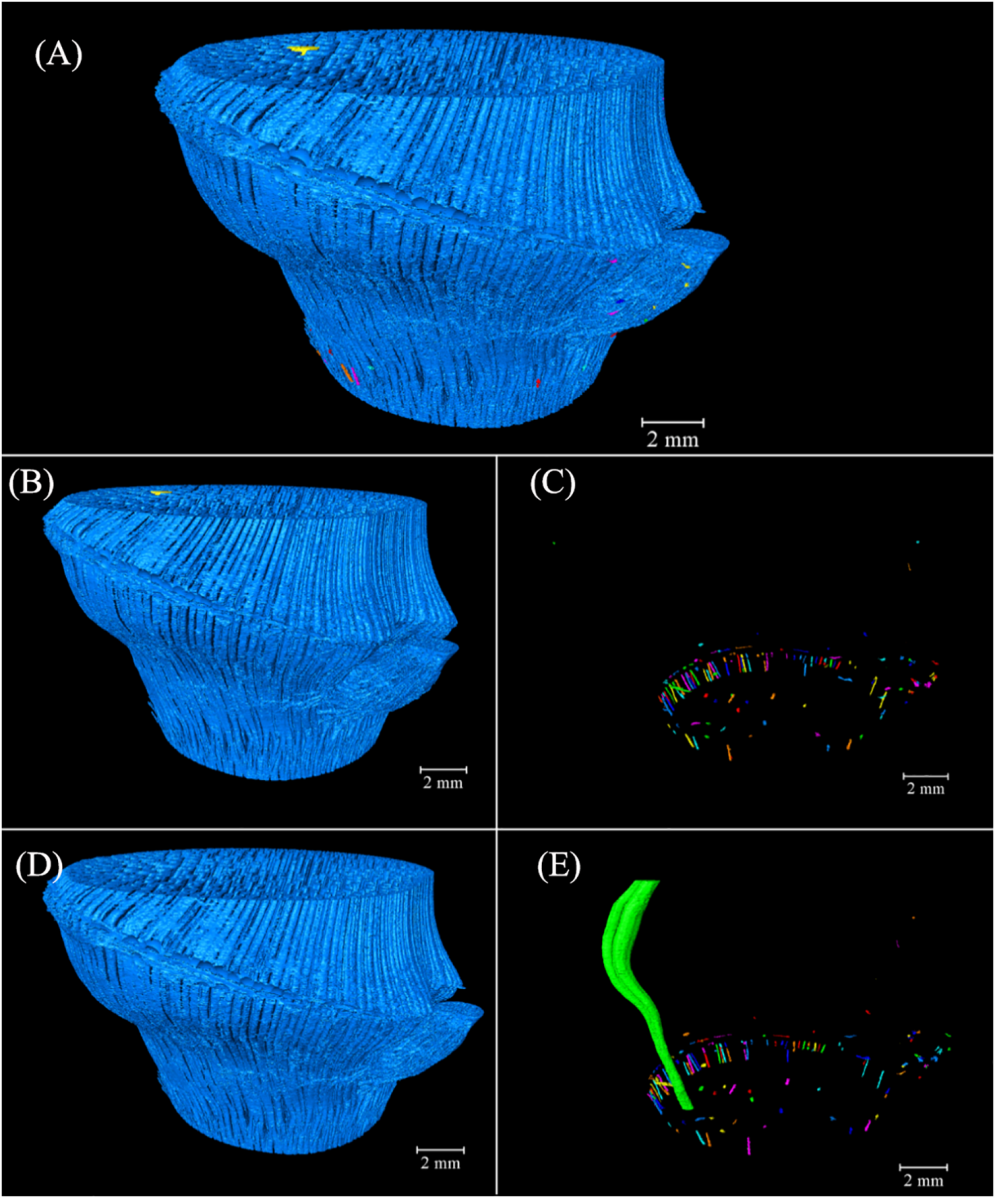
Spatial arrangement and orientation of fibers in the 3D model of *Chimonobambusa tumidissinoda* after label analysis: **(A)** The fibers; **(B)** Connected fibers; **(C)** Disconnected fibers; **(D)** The largest fiber volume; **(E)** The fibers without the largest volume.

Image labeling provided valuable 3D insights into the complex fibers of the BN, allowing for the differentiation of volume units through the use of distinct colors (**Figure 9A**). Notably, 483.56 mm³ of the connected fibers accounted for 99.95% of 483.79 mm³ of the total fibers in the BN (**Figure 9B**). The connectivity of the fibers was higher than that of the conducting tissue because of the larger area of fibers in the cross-section, which enhanced segmentation during image processing. Some branching of the VB under the bottom of the BN got into the sheath scar and stopped growing, possibly giving rise to the disconnected fibers distributed near the sheath scar. Small-sized fibers in the branch possibly also led to a disconnection of the fibers (**Figure 9C**). The largest fibers, with 481.22 mm³ comprised 99.47% of the fibers in the BN (**Figure 9D**), with the fibers of the culm and branch twined and connected to form whole fibers. However, 0.53% of the fibers were unconnected with the largest fibers. Most of them were like disconnected fibers (**Figure 9E**) and possibly connected to other bamboo parts. While the study unveiled the interrelation of VBs between the branch and the culm, the high connectivity of fibers and conducting tissue within the BN necessitates further observation to understand the spatial relationships among VBs exhibiting diverse anatomical characteristics in the cross-sections.

## 4 Conclusions

This study employed µCT scanning and 3D reconstruction techniques to observe and analyze the anatomical structure of branched bamboo. The morphology, quantity, frequency, size, and tissue proportion of the VB in a BN were affected by its branch, which caused the gradient structure of bamboo. The 3D visualization of the fibers and conducting tissue was successfully realized, providing real evidence of the structure between the culm and the branch of the BN. Fibers and conducting tissue had similar morphology, yet exhibited distinct variations in thickness patterns, allowing VBs to perform the dual functions of water transport and mechanical support simultaneously. The 3D structure of the VB in the BN provided a new perspective for understanding the performance of the bamboo node, nodal ridge was a weak point in the mechanical properties of BN.

## Data availability statement

The original contributions presented in the study are included in the article/**Supplementary Material**. Further inquiries can be directed to the corresponding author.

## Author contributions

SL: Software, Writing – original draft. QY: Software, Visualization, Methodology, Writing – original draft. YW: Writing – review & editing. LS: Formal Analysis, Software, Visualization, Writing – review & editing. SY: Conceptualization, Methodology, Validation, Writing – review & editing. XL: Formal Analysis, Investigation, Writing – review & editing. QM: Data curation, Resources, Writing – review & editing. ZC: Software, Writing – original draft.

## Supplementary material

Supplementary Figure 1 Cross-sectional images of some bamboo nodes. (The yellow arrow indicates the position of the cross-section along the height of the bamboo node). Sample 1: the bottom of the BN (left); the nodal ridge (right). Sample 2: near the diaphragm (left); the nodal ridge (right). Sample 3: the bottom of the BN (left); the nodal ridge (right). Sample 4: the diaphragm (left); the nodal ridge (right). (Note: the brighter regions in the Sample 2 and 3 were caused by higher moisture content).

## References

[B1] Ahmad M. Kamke F. (2005). Analysis of Calcutta bamboo for structural composite materials: physical and mechanical properties. Wood Sci. Technol. 39, 448–459. doi: 10.1007/s00226-005-0016-y

[B2] Amada S. Untao S. (2001). Fracture properties of bamboo. Compos. Part B Eng. 32 (5), 451–459. doi: 10.1016/S1359-8368(01)00022-1

[B3] Bouxsein M. L. Boyd S. K. Christiansen B. A. Guldberg R. E. Jepsen K. J. Müller R. (2010). Guidelines for assessment of bone microstructure in rodents using micro–computed tomography. J. Bone Miner. Res. 25 (7), 1468–1486. doi: 10.1002/jbmr.141 20533309

[B4] Chen G. Luo H. Wu S. Guan J. Luo J. Zhao T. (2018). Flexural deformation and fracture behaviors of bamboo with gradient hierarchical fibrous structure and water content. Compos. Sci. Technol. 157, 126–133. doi: 10.1016/j.compscitech.2018.01.034

[B5] Chen M. Ye L. Li H. Wang G. Chen Q. Fang C.. (2020). Flexural strength and ductility of moso bamboo. Constr. Build. Mater. 246, 118418. doi: 10.1016/j.conbuildmat.2020.118418

[B6] Chen S. M. Zhang S. C. Gao H. L. Wang Q. Zhou L. Zhao H. Y.. (2023). Mechanically robust bamboo node and its hierarchically fibrous structural design. Natl. Sci. Rev. 10 (2), nwac195. doi: 10.1093/nsr/nwac195 36817831 PMC9935994

[B7] Cui J. Qin Z. Masic A. Buehler M. J. (2020). Multiscale structural insights of load bearing bamboo: A computational modeling approach. J. Mech. Behav. Biomed. 107, 103743. doi: 10.1016/j.jmbbm.2020.103743 32364947

[B8] Ding Y. L. Fan R. W. Huang J. S. (2000). Development and ultrastructure of the phloem ganglion in bamboo node. Acta Botanica Sinica. 10), 1009–1013.

[B9] Ding Y. L. Liese W. (1995). On the structure of bamboo. J. Bamboo Res. 01), 24–32.

[B10] Fang C. H. Jiang Z. H. Sun Z. J. Liu H. R. Zhang X. B. Zhang R.. (2018). An overview on bamboo culm flattening. Constr. Build. Mater. 171, 65–74. doi: 10.1016/j.conbuildmat.2018.03.085

[B11] Ghavami K. Rodrigues C. S. Paciornik S. (2003). Bamboo: functionally graded composite material. Asian J. Civ. Eng. (Build. Hous.). 4, 1–10.

[B12] Grosser D. Liese W. (1971). On the anatomy of Asian bamboos with special reference to their vascular bundles. Wood Sci. Technol. 5 (4), 290–312. doi: 10.1007/BF00365061

[B13] Hamdan H. U. M. K. Anwar U. M. K. Zaidon A. Tamizi M. M. (2009). Mechanical properties and failure behaviour of Gigantochloa scortechinii. J. Trop. For. Sci. 21 (4), 336–344.

[B14] Huang P. Chang W. S. Ansell M. P. Chew Y. J. Shea A. (2015). Density distribution profile for internodes and nodes of Phyllostachys edulis (Moso bamboo) by computer tomography scanning. Constr. Build. Mater. 93, 197–204. doi: 10.1016/j.conbuildmat.2015.05.120

[B15] Huang P. Chang W. S. Ansell M. P. John C. Y. Shea A. (2017). Porosity estimation of Phyllostachys edulis (Moso bamboo) by computed tomography and backscattered electron imaging. Wood Sci. Technol. 51 (1), 11–27. doi: 10.1007/s00226-016-0865-6

[B16] Kanzawa E. Aoyagi S. Nakano T. (2011). Vascular bundle shape in cross-section and relaxation properties of Moso bamboo (Phyllostachys pubescens). Mater. Sci. Eng.: C. 31 (5), 1050–1054. doi: 10.1016/j.msec.2011.03.004

[B17] Kappel R. Mattheck C. Bethge K. Tesari I. (2004). Bamboo as a composite structure and its mechanical failure behaviour. WIT. Ecol. Envir. 73, 9. doi: 10.2495/dn040291

[B18] Khalil H. A. Bhat I. U. H. Jawaid M. Zaidon A. Hermawan D. Hadi Y. S. (2012). Bamboo fibre reinforced biocomposites: A review. Mater Design. 42, 353–368. doi: 10.1016/j.matdes.2012.06.015

[B19] Kraehmer H. (2017). On vascular bundle modifications in nodes and internodes of selected grass species. Sci. Agr. Bohem. 48 (3), 112–121. doi: 10.1515/sab-2017-0018

[B20] Landis E. N. Keane D. T. (2010). X-ray microtomography. Mater. Charact. 61 (12), 1305–1316. doi: 10.1016/j.matchar.2010.09.012

[B21] Lee A. W. Liu Y. (2003). Selected physical properties of commercial bamboo flooring. For. Prod. J. 53 (6), 23.

[B22] Li S. Yang S. Shang L. Liu X. Ma J. Ma Q.. (2021). 3D visualization of bamboo node’s vascular bundle. Forests. 12 (12), 1799. doi: 10.3390/f12121799

[B23] Liu L. Luan Y. Fang C. Hu J. Chang S. Fei B. (2023). Structural characteristics of reaction tissue in plants. Plants. 12 (8), 1705. doi: 10.3390/plants12081705 37111927 PMC10146549

[B24] Odgaard A. Gundersen H. J. G. (1993). Quantification of connectivity in cancellous bone, with special emphasis on 3-D reconstructions. Bone. 14 (2), 173–182. doi: 10.1016/8756-3282(93)90245-6 8334036

[B25] Palombini F. L. Kindlein W. Jr. de Oliveira B. F. de Araujo Mariath J. E. (2016). Bionics and design: 3D microstructural characterization and numerical analysis of bamboo based on X-ray microtomography. J. Mater. Charact. 120, 357–368. doi: 10.1016/j.matchar.2016.09.022

[B26] Palombini F. L. Nogueira F. M. Kindlein W. Paciornik S. de Araujo Mariath J. E. de Oliveira B. F. (2020). Biomimetic systems and design in the 3D characterization of the complex vascular system of bamboo node based on X-ray microtomography and finite unit analysis. J. Mater. Res. 35 (8), 842–854. doi: 10.1557/jmr.2019.117

[B27] Peng G. Jiang Z. Liu X. E. Fei B. Yang S. Qin D.. (2014). Detection of complex vascular system in bamboo node by X-ray μCT imaging technique. Holzforschung. 68 (2), 223–227. doi: 10.1515/hf-2013-0080

[B28] Shao Z. P. Fang C. H. Huang S. X. Tian G. L. (2010a). Tensile properties of Moso bamboo (Phyllostachys pubescens) and its components with respect to its fiber-reinforced composite structure. Wood Sci. Technol. 44 (4), 655–666. doi: 10.1007/s00226-009-0290-1

[B29] Shao Z. P. Zhou L. Liu Y. M. Wu Z. M. Arnaud C. (2010b). Differences in structure and strength between internode and node sections of moso bamboo. J. Trop. For. Sci. 22 (2), 133–138.

[B30] Silin D. Tomutsa L. Benson S. M. Patzek T. W. (2011). Microtomography and pore-scale modeling of two-phase fluid distribution. Transport porous Med. 86 (2), 495–515. doi: 10.1007/s11242-010-9636-2

[B31] Song J. Gao L. Lu Y. (2017). *In situ* mechanical characterization of structural bamboo materials under flexural bending. Exp. Tech. 41, 565–575. doi: 10.1007/s40799-017-0202-5

[B32] Sun Y. Sills R. B. Hu X. Seh Z. W. Xiao X. Xu H.. (2015). A bamboo-inspired nanostructure design for flexible, foldable, and twistable energy storage devices. Nano Lett. 15 (6), 3899–3906. doi: 10.1021/acs.nanolett.5b00738 26011653

[B33] Taylor D. Kinane B. Sweeney C. Sweetnam D. O’Reilly P. Duan K. (2015). The biomechanics of bamboo: investigating the role of the nodes. Wood Sci. Technol. 49 (2), 345–357. doi: 10.1007/s00226-014-0694-4

[B34] Wang X. Ren H. Zhang B. Fei B. Burgert I. (2012). Cell wall structure and formation of maturing fibres of moso bamboo (Phyllostachys pubescens) increase buckling resistance. J. R. Soc Interface 9 (70), 988–996. doi: 10.1098/rsif.2011.0462 21920959 PMC3306637

[B35] Wang F. Shao Z. Wu Y. (2014). The toughness contribution of bamboo node to the mode I interlaminar fracture toughness of bamboo. Wood Sci. Technol. 48 (6), 1257–1268. doi: 10.1007/s00226-013-0591-2

[B36] Wang F. Shao Z. (2020). Study on the variation law of bamboo fibers’ tensile properties and the organization structure on the radial direction of bamboo stem. Ind. Crop Prod. 152, 112521. doi: 10.1016/j.indcrop.2020.112521

[B37] Wegst U. G. (2008). Bamboo and wood in musical instruments. Annu. Rev. Mater. Res. 38, 323–349. doi: 10.1146/annurev.matsci.38.060407.132459

[B38] Xian X. Xian D. (1990). The relationship of microstructure and mechanical properties of bamboo. J. Bamboo Res. 9 (3), 10–123.

[B39] Xiang E. Yang S. Cao C. Liu X. Peng G. Shang L.. (2021). Visualizing complex anatomical structure in bamboo nodes based on x-ray microtomography. J. Renew. Mater. 9 (9), 1531. doi: 10.32604/jrm.2021.015346

[B40] Xiong W. Y. Qiao S. Y. Li Y. F. (1980). The anatomical structure of culm of Phyllostachys pubescens Mazel ex H.de Lehaie. J. Integr. Plant Biol. 04), 343–348+416-417.

[B41] Yamaji N. Ma J. (2017). Node-controlled allocation of mineral elements in Poaceae. Curr. Opin. Plant Biol. 39, 18–24. doi: 10.1016/j.pbi.2017.05.002 28558362

[B42] Yu W. J. Jiang Z. H. Ye K. L. (2002). Characteristics research of bamboo and its development. World For. Res. 02), 50–55.

[B43] Yuan J. Chen L. Mi B. Lei Y. Yan L. Fei B. (2023). Synergistic effects of bamboo cells during shrinkage process. Ind. Crop Prod. 193, 116232. doi: 10.1016/j.indcrop.2022.116232

[B44] Zhang X. Li J. Yu Z. Yu Y. Wang H. (2017a). Compressive failure mechanism and buckling analysis of the graded hierarchical bamboo structure. J. Mater. Sci. 52 (12), 6999–7007. doi: 10.1007/s10853-017-0933-9

[B45] Zhang Q. Liang G. Y. Zhang X. C. Zhang W. B. Wang S. T. Bin L. C.. (2017b). The effect of structural properties of moso bamboo on the open groove. J. bamboo Res. 36 (1), 59–63.

[B46] Zou M. Song J. Xu S. Liu S. Chang Z. Merodio J. (2018). Bionic design of the bumper beam inspired by the bending and energy absorption characteristics of bamboo. Appl. Bionics Biomech. 2018, 1–12. doi: 10.1155/2018/8062321 PMC6305026 30627217

[B47] Zou M. Xu S. Wei C. Wang H. Liu Z. (2016). A bionic method for the crashworthiness design of thin-walled structures inspired by bamboo. Thin-Walled Struct. 101, 222–230. doi: 10.1016/j.tws.2015.12.023

