# Supplementary figures and images for: Three-dimensional visualization of the vascular bundle in a branched bamboo node
Source: Front Plant Sci. 2023 Oct 25;14:1256772. doi: 10.3389/fpls.2023.1256772 (PMC10634429; doi:10.3389/fpls.2023.1256772)

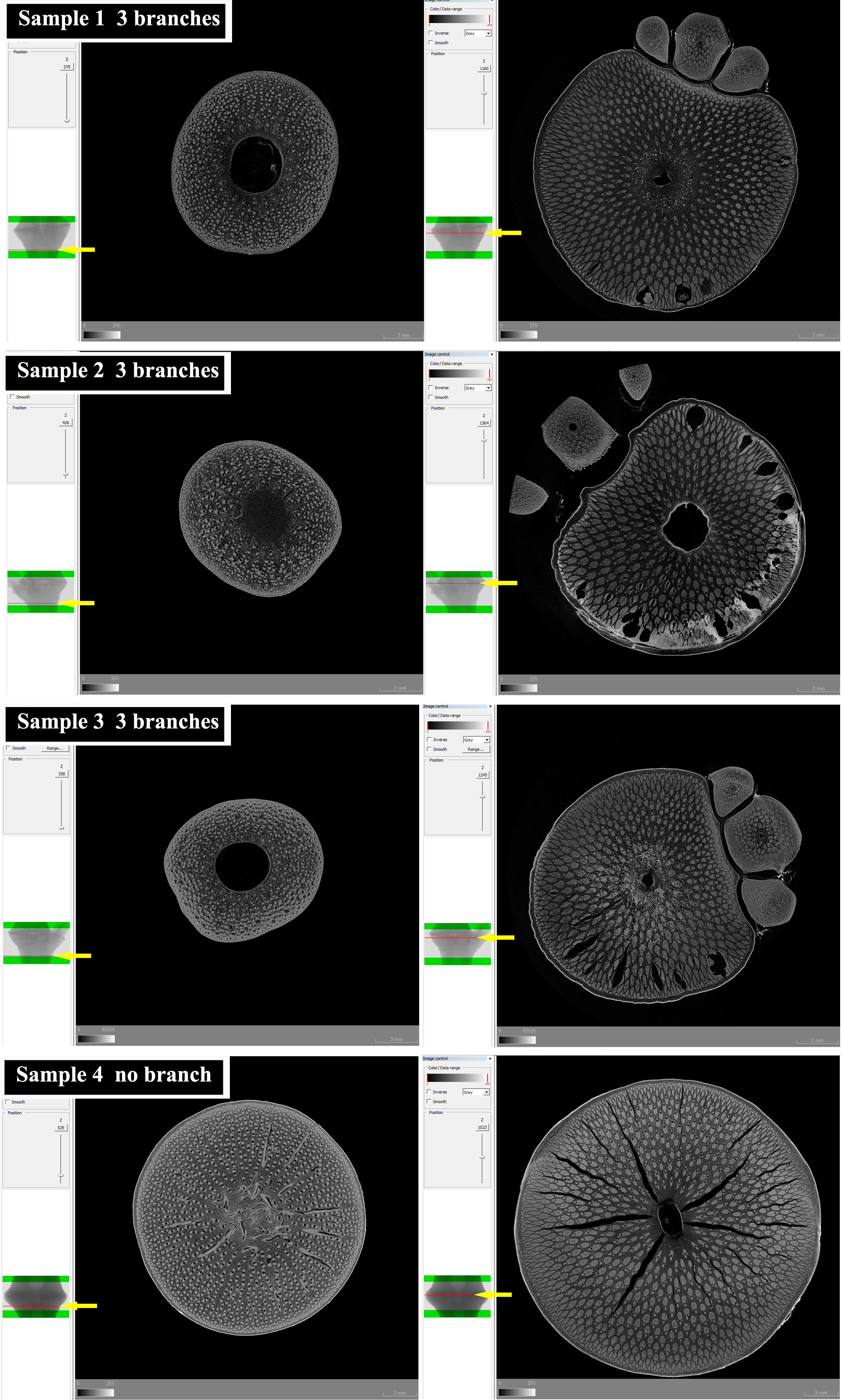

Supplement: Supplementary Figure 1 — Cross-sectional images of some bamboo nodes. (The yellow arrow indicates the position of the cross-section along the height of the bamboo node). Sample 1: the bottom of the BN (left); the nodal ridge (right). Sample 2: near the diaphragm (left); the nodal ridge (right). Sample 3: the bottom of the BN (left); the nodal ridge (right). Sample 4: the diaphragm (left); the nodal ridge (right). (Note: the brighter regions in the Sample 2 and 3 were caused by higher moisture content). [file Image_1.tif]
